# Bioinformatics analysis constructs potential ferroptosis-related ceRNA network involved in the formation of intracranial aneurysm

**DOI:** 10.3389/fncel.2022.1016682

**Published:** 2022-10-13

**Authors:** Huaxin Zhu, Jiacong Tan, Zhihua Wang, Zhiwu Wu, Wu Zhou, Zhixiong Zhang, Meihua Li, Yeyu Zhao

**Affiliations:** Department of Neurosurgery, The First Affiliated Hospital of Nanchang University, Nanchang, Jiangxi, China

**Keywords:** intracranial aneurysm, ferroptosis, ceRNA network, gene expression omnibus, bioinformatics (genome and proteome) databases

## Abstract

**Background:**

Intracranial aneurysm (IA) causes more than 80% of nontraumatic subarachnoid hemorrhages (SAHs). The mechanism of ferroptosis involved in IA formation remains unclear. The roles played by competitive endogenous RNA (ceRNA) regulation networks in many diseases are becoming clearer. The goal of this study was to understand more fully the ferroptosis-related ceRNA regulation network in IA.

**Materials and methods:**

To identify differentially expressed genes (DEGs), differentially expressed miRNAs (DEMs), and differentially expressed lncRNAs (DELs) across IA and control samples, the GEO datasets GSE122897 and GSE66239 were downloaded and analyzed with the aid of R. Ferroptosis DEGs were discovered by exploring the DEGs of ferroptosis-related genes of the ferroptosis database. Potentially interacting miRNAs and lncRNAs were predicted using miRWalk and StarBase. Enrichment analysis was also performed. We utilized the STRING database and Cytoscape software to identify protein-protein interactions and networks. DAB-enhanced Prussian blue staining was used to detect iron in IA tissues.

**Results:**

Iron deposition was evident in IA tissue. In all, 30 ferroptosis DEGs, 5 key DEMs, and 17 key DELs were screened out for constructing a triple regulatory network. According to expression regulation of DELs, DEMs, and DEGs, a hub triple regulatory network was built. As the functions of lncRNAs are determined by their cellular location, PVT1-hsa-miR-4644-SLC39A14 ceRNA and DUXAP8-hsa-miR-378e/378f-SLC2A3 ceRNA networks were constructed.

**Conclusion:**

CeRNA (PVT1-hsa-miR-4644-SLC39A14 and DUXAP8-hsa-miR-378e/378f-SLC2A3) overexpression networks associated with ferroptosis in IA were established.

## Introduction

Intracranial aneurysm (IA) is a common cerebrovascular disease, with an incidence of about 1–2%, and saccular aneurysm is its most common type (Brown and Broderick, 2014; Etminan and Rinkel, 2016). In the Chinese population aged 35–75 years, the prevalence of IA is as high as 7% (Chen et al., 2018). More than 80% of nontraumatic subarachnoid hemorrhages (SAHs) are caused by IA rupture (Kassell et al., 1990; Koo et al., 2022). Aneurysmal SAHs have high mortality and disability rates, and are associated with devastating complications, such as delayed cerebral ischemia, seizures, and cerebral vasospasm (CVS), which can lead to poor prognosis and a heavy burden on society (Chalouhi et al., 2013; Chaudhry et al., 2021). IA is currently thought to be related to many factors, including age, hypertension, a genetic predisposition, hemodynamic changes, and environmental factors (Kwon, 2019). Despite decades of research on IA, the pathophysiological mechanisms underlying aneurysm formation, progression, and rupture remain unknown (Chalouhi et al., 2016; Pascale et al., 2020).

Stockwell was the first to propose the concept of ferroptosis, a type of cell death that differs from apoptosis, necroptosis, and autophagy (Dixon et al., 2012). Its occurrence has specific characteristics and regulatory genes or proteins. Morphologically, mitochondrial volume is reduced and mitochondrial cristae are reduced or absent (Jang et al., 2021). The inability to metabolize lipid peroxides reflects glutathione depletion and diminished glutathione peroxidase-4 (GPX4) activity, promoting ferroptosis (Imai et al., 2017). Immune cells release pro-inflammatory mediators including high mobility group protein B1 (HMGB1) when proteins with damage-associated molecular patterns (DAMPs) are released (Wu et al., 2021). The pathophysiology of cardiovascular disorders such as cardiomyopathy, atherosclerosis, and abdominal aortic aneurysms (AAAs) is complicated by ferroptosis (Sawada et al., 2015; Chen et al., 2019; Fang et al., 2019; Ouyang et al., 2021). HMGB1 is highly expressed in IA walls, particularly in ruptured IA tissue, and in patients with intracranial aneurysmal SAHs; increased HMGB1 levels are positively correlated with poor prognosis and cerebral vasospasm (Zhang et al., 2016; Chaudhry et al., 2018). However, the relationship between IA formation and ferroptosis remains unclear, and more research is needed to confirm the mechanism.

Non-coding RNAs, such as long non-coding RNAs (lncRNAs), short microRNAs (miRNAs), and circular RNAs (circRNAs), account for 95% of all eukaryotic RNAs (Hombach and Kretz, 2016). MiRNAs are small RNA molecules of about 22 nucleotides that regulate gene expression by degrading or inhibiting the transcription of target messenger RNA (mRNA). The functions affected include cell proliferation, differentiation, apoptosis, and disease onset and progression (Bartel, 2004; Zhang et al., 2019; Wittstatt et al., 2020). MiRNA-29a controls the mitochondrial apoptotic pathway, while miRNA-513b-5p targets the COL1A1 and COL1A2 proteins involved in IA formation and rupture (Zhao et al., 2018; Zheng et al., 2021). The lncRNAs are more than 200 nucleotides in length but do not have protein-coding capacity (Wilusz, 2016). The aberrant expression of lncRNAs in IA suggests a possible role in its pathogenesis, and various studies have reported the importance of lncRNAs in IA (Li et al., 2020; Pan W. et al., 2021; Pan Y. B. et al., 2021). Both miRNAs and lncRNAs also play critical roles in ferroptosis regulation. The lncRNAs are longer than 200 nucleotides but do not encode proteins. The lncRNA ZFAS1 promotes lung fibroblast-to-myofibroblast transition and ferroptosis through the miR-150-5p/SLC38A1 axis, whereas the lncRNA PVT1 controls ferroptosis through miR-214-mediated TFR1 and p53 (Lu et al., 2020; Yang et al., 2020). However, ferroptosis regulation by the lncRNA-miRNA-mRNA axis has rarely been investigated in IA.

Thus, we used staining to confirm iron deposition in IA and concluded that ferroptosis may be involved in IA onset and progression. We compared IA and normal tissues; we performed data mining and analysis to detect differentially expressed genes (DEGs), differentially expressed miRNAs (DEMs), and differentially expressed lncRNAs (DELs). An important lncRNA-miRNA-mRNA axis was discovered by constructing a ceRNA network using key DEGs, DEMs, and DELs. To better understand further the molecular causes of IA, we built a possible ferroptosis lncRNA-miRNA-mRNA regulation network.

## Materials and methods

### Superficial temporal artery and intracranial aneurysm tissues

Intracranial aneurysm tissue was obtained from patients undergoing surgical IA clipping, and STA tissue was obtained from patients with brain trauma who underwent removal of intracranial hematomas. All tissues were immediately fixed with 4% (v/v) paraformaldehyde and embedded in paraffin. All patients gave written informed consent prior to surgery. The First Affiliated Hospital of Nanchang University Ethics Committee approved the work.

### Diaminobenzidine-enhanced Prussian blue staining

The Prussian Blue Iron Stain Kit, enhanced with diaminobenzidine (DAB), was obtained from Beijing Solarbio Science and Technology Co., Ltd. (Beijing, China). Paraffin tissues were dewaxed and hydrated with xylene and ethanol. The tissues were first exposed to Prussian blue for 30 min at 37°C, and then to the DAB chromogenic solution for 15 min at 37°C; they were counterstained for 5 min in hematoxylin staining solution, rendered transparent using xylene, dehydrated through graded ethanol baths, and mounted with neutral gum.

### Data collection and preprocessing

**FIGURE 1:**
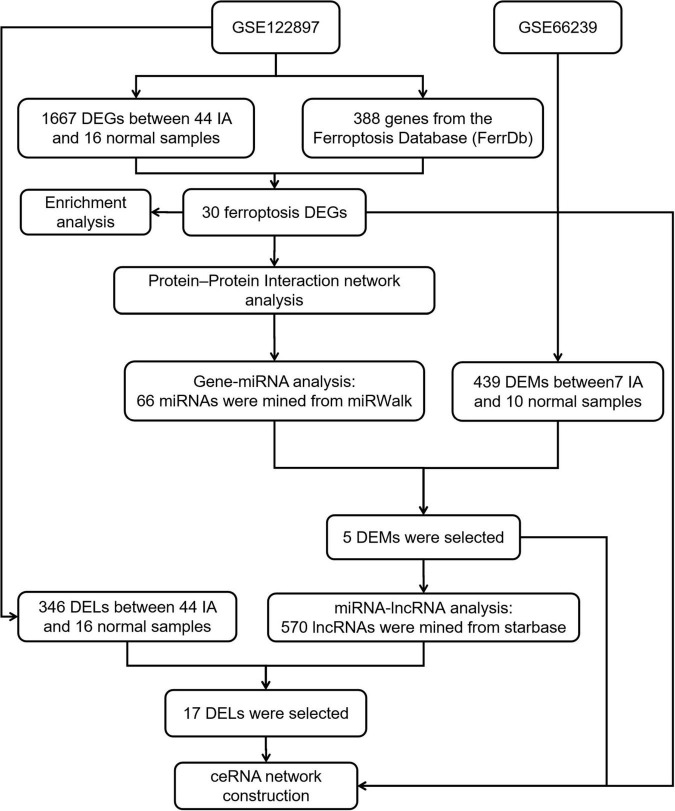
A flowchart showing how the lncRNA–miRNA–mRNA regulatory network of an intracranial aneurysm (IA) was constructed.

Transcriptome information on IA patients was gathered from the Gene Expression Omnibus (GEO database at https://www.ncbi.nlm.nih.gov/geo/). IA-related datasets, GSE122897 and GSE66239, were downloaded from the GEO database and included in the analysis. The GSE122897 dataset, which contained 44 IA and 16 control samples, was used for expression profiling. GSE66239 is a microRNA dataset that contained 7 IA and 10 control samples. The workflow is depicted in Figure 1.

### Differentially expressed gene, miRNA, and long non-coding RNA analysis

R package DESeq2 (1.26.0: The R foundation, Vienna, Austria) was used to analyze IA and control samples for obtaining DEGs, DEMs, and DELs. DEGs and DELs were obtained from GSE122897, while DEMs were obtained from GSE66239. Log2(FC) > 1.0 and *P*-value < 0.05 were considered the thresholds for DEGs, DEMs, and DELs. The results were displayed using volcano plots. We also used a dataset from the Ferroptosis database (FerrDb)^1^ to find ferroptosis DEGs by intersecting ferroptosis genes with DEGs.

### Functional and pathway enrichment analyses

For the GO and KEGG enrichment analyses, the R package clusterProfiler (3.14.3) was utilized. The findings were downloaded using the R packages dplyr (version 1.0.7) and ggplot2 (version 3.3.5). We uploaded the ferroptosis DEGs to Metascape, an online tool for gene function annotation analysis (Li et al., 2014).^2^

### Protein–protein interaction network analysis

Using String (version 11.5)^3^, a PPI network for ferroptosis DEGs was created. PPI networks and modules playing essential roles in ferroptosis were further examined using Cytoscape (version 3.8.2).

### Gene-miRNA interaction networks

To determine the crucial miRNAs and to develop DEG-miRNA interaction networks, we utilized miRWalk (version 3).^4^ To ensure the correctness of the results, we combined the TargetScan and miRWalk results. Targeted miRNAs were intersected with DEMs from GSE66240 to identify key miRNAs. The DEG-miRNA network was visualized using Cytoscape.

### miRNA–lncRNA interaction networks

Upstream lncRNAs for key miRNAs were identified using StarBase (Li et al., 2014).^5^ Potential lncRNAs were intersected with DELs from GSE122897 to identify key lncRNAs. The key DEGs, DEMs, and DELs worked together to construct the triple regulatory network, and identified key ceRNAs through their expression regulation. Target sites were predicted using StarBase. DEL sequences were retrieved from LNCipedia^6^; the lncLocator database^7^ was used to determine DEL cellular localizations. The networks were visualized using Cytoscape.

### Immunohistochemistry

Paraffin tissues were dewaxed and rehydrated, immersed in EDTA solution (1 mM, pH 8.0), heated at 95°C for 1 h, treated with 3% hydrogen peroxide, incubated with 10% goat serum, and incubated overnight at 4°C using anti-SLC39A14 (Proteintech, Chicago, IL, USA) and anti-SLC2A3 (Affinity Biosciences, Nanjing, China), respectively. After incubation, slides were treated with biotinylated IgG and horseradish peroxidase-labeled streptomycin, respectively. Finally, the slides were stained with 3,3′-diaminobenzidine (DAB) and restained with hematoxylin. Images were taken under a microscope (ZEISS, Oberkochen, Germany).

### Statistical analysis

R (version 4.1.0; the R foundation) was used for data preprocessing; DEG, DEM, and DEL screening; and enrichment analysis. Metascape was employed for functional annotation analysis. String was used to identify PPI networks. Potential miRNA and lncRNA interactions were mined using miRWalk and StarBase, respectively. The networks were visualized using Cytoscape. *P* < 0.05 was taken to indicate statistical significance.

## Results

### Ferroptosis differentially expressed genes and iron in tissues

**FIGURE 2:**
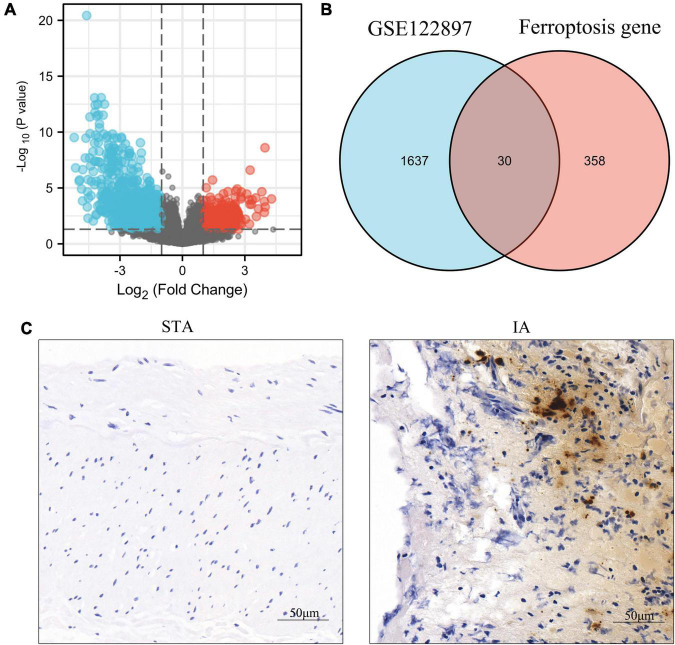
Identification of ferroptosis-related differentially expressed genes (DEGs) in GSE122897 and detection of iron in tissues. **(A)** Volcano plot of DEGs. **(B)** Venn diagram of ferroptosis DEGs. **(C)** Iron staining within superficial temporal artery (STA) and intracranial aneurysm (IA) tissues.

We recovered 1,667 DEGs of the GSE122897 dataset based on a *p*-values 0.05 and | log2FC| > 1. Of these, 483 evidenced upregulation and 1,184 downregulation (Figure 2A and Supplementary Table 1). The ferroptosis database (FerrDb) yielded 388 ferroptosis-related genes, which were overlapped with GSE122897 genes to locate ferroptosis DEGs (Figure 2B); 20 upregulated and 10 downregulated genes were identified (Table 1). Using the online FerrDb tool, the ferroptosis DEGs were further classified as ferroptosis drivers, suppressors, and markers (Table 2). DAB-enhanced Prussian blue staining revealed greater iron deposition in IA tissues compared to STA tissues (Figure 2C).

**TABLE 1:** Ferroptosis differentially expressed genes (DEGs) of intracranial aneurysm (IA).

Gene symbol	Fold change	*P*-value	Gene title
**Upregulated genes**			
ABCC1	1.14	<0.001	ATP binding cassette subfamily C member 1
AQP3	1.03	0.013	Aquaporin 3
CDKN2A	1.40	0.031	Cyclin dependent kinase inhibitor 2A
CP	1.34	0.012	Ceruloplasmin
CXCL2	1.30	0.011	C–X–C motif chemokine ligand 2
DDIT4	1.11	0.016	DNA damage inducible transcript 4
HIF1A	1.02	0.017	Hypoxia inducible factor 1 subunit alpha
IL6	1.94	0.005	Interleukin 6
NNMT	1.38	0.013	Nicotinamide *N*-methyltransferase
NOX4	1.12	0.035	NADPH oxidase 4
PLIN2	1.72	0.003	Perilipin 2
PTGS2	1.20	0.011	Prostaglandin-endoperoxide synthase 2
RGS4	1.22	0.011	Regulator of G protein signaling 4
SAT1	1.04	0.033	Spermidine/spermine N1-acetyltransferase 1
SLC2A3	1.01	0.012	Solute carrier family 2 member 3
SLC39A14	1.32	0.007	Solute carrier family 39 member 14
TNFAIP3	1.49	0.004	TNF alpha induced protein 3
VDR	1.53	0.028	Vitamin D receptor
VEGFA	1.31	0.019	Vascular endothelial growth factor A
ZFP69B	1.01	0.003	ZFP69 zinc finger protein B
**Downregulated genes**			
AQP5	−1.98	0.022	Aquaporin 5
ATP6V1G2	−2.53	0.000	ATPase H+ transporting V1 subunit G2
FNDC5	−1.05	0.031	Fibronectin type III domain containing 5
GLS2	−1.06	0.010	Glutaminase 2
GPT2	−1.39	0.003	Glutamic–pyruvic transaminase 2
MT3	−3.61	<0.001	Metallothionein 3
PRKCA	−1.00	0.003	Protein kinase C alpha
PSAT1	−1.93	0.001	Phosphoserine aminotransferase 1
SLC2A12	−2.34	<0.001	Solute carrier family 2 member 12
SLC7A11	−1.35	0.013	Solute carrier family 7 member 11

**TABLE 2:** The ferroptosis differentially expressed genes (DEGs) were divided into ferroptosis driver, suppressor, and marker.

Driver	Suppressor	Marker
NOX4, ABCC1, SLC39A14, TNFAIP3, SAT1, GLS2, CDKN2A, PRKCA, AQP5, AQP3, and HIF1A IL6	CP, VDR, PLIN2, FNDC5, HIF1A, and SLC7A11	SLC2A3, PTGS2, CXCL2, MT3, VEGFA, RGS4, PSAT1, SLC2A12, GPT2, NNMT, DDIT4, ZFP69B, ATP6V1G2, IL6, and SLC7A11

### Enrichment analysis and protein–protein interaction network analysis of ferroptosis differentially expressed genes

**FIGURE 3:**
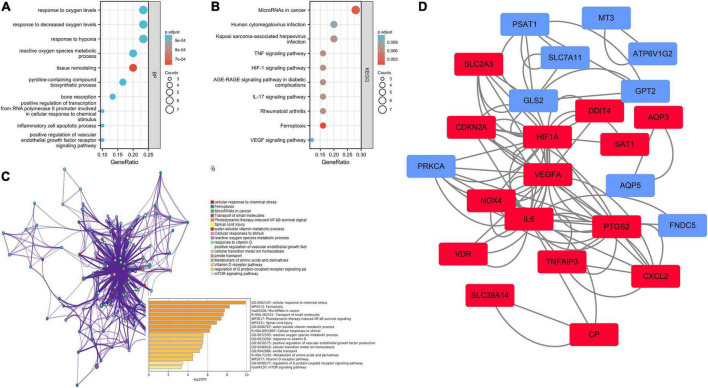
Enrichment analysis and the protein–protein interaction (PPI) network of the ferroptosis DEGs. **(A)** Gene set enrichment analysis of Gene Ontology biological process (GO-BP). **(B)** Gene set enrichment analysis of Kyoto Encyclopedia of Genes and Genomes (KEGG). **(C)** Network and bar chart of the enrichment results drawn by Metascape. **(D)** Construction of the PPI network. Navy-blue signifies downregulation and red upregulation.

The biological functions of ferroptosis DEGs were investigated using enrichment analysis employing the GO-BP (Figure 3A) and KEGG (Figure 3B) gene sets (Supplementary Table 2). Tissue remodeling, response to hypoxia, positive regulation of the vascular endothelial growth factor receptor signaling pathway, the response to decreased oxygen levels, the response to oxygen levels, inflammatory cell apoptotic processes, and reactive oxygen species metabolic processes were all significantly activated on GO-BP enrichment analysis. Ferroptosis and microRNAs may be involved in the IA process, as revealed by KEGG pathway enrichment analysis. Enrichment results drawn by Metascape were similar (Figure 3C). To create PPI networks, all ferroptosis DEGs were entered into the String database. The results were downloaded and visualized using Cytoscape (Figure 3D).

### Further miRNA interactions and mining

**FIGURE 4:**
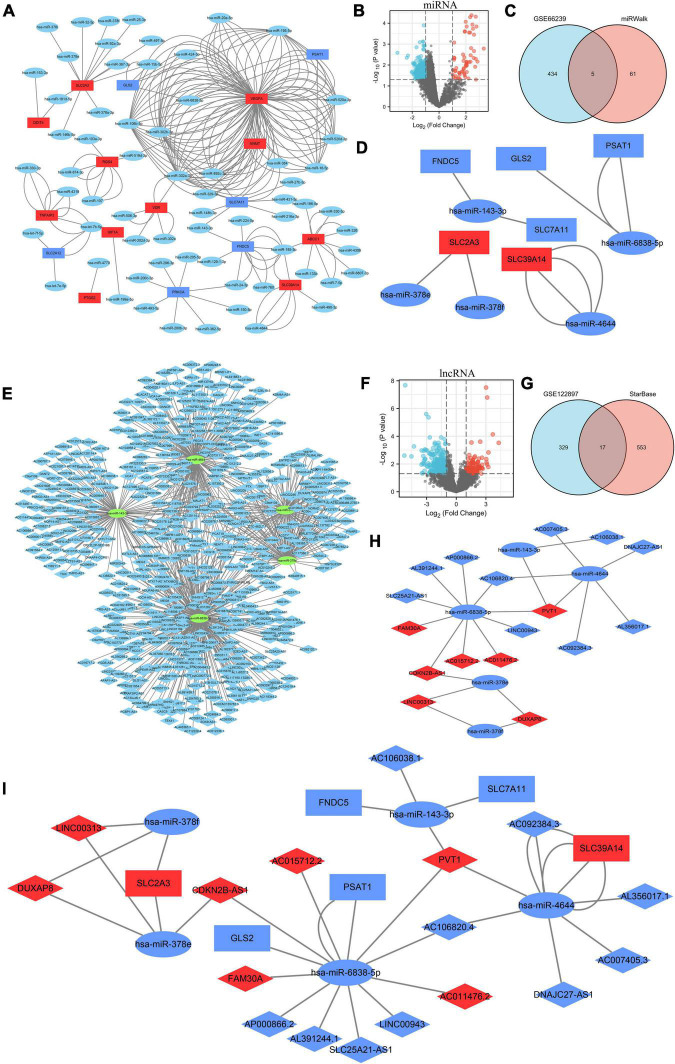
Construction of the triple regulatory network of differentially expressed lncRNAs (DELs), differentially expressed miRNAs (DEMs), and ferroptosis DEGs. **(A)** The interaction network between ferroptosis DEGs and the targeted miRNAs. **(B)** Volcano plot of DEMs in GSE66240. **(C)** Venn diagram of DEMs and potentially targeted miRNAs encoding ferroptosis DEGs. **(D)** Interaction network between the overlapped DEMs and the ferroptosis DEGs. **(E)** Interaction network between the overlapped DEMs and the targeted lncRNAs. **(F)** Volcano plot for DELs of GSE122897. **(G)** Venn diagram of DELs and the potentially targeted lncRNAs of the overlapped DELs. **(H)** Interaction network of the overlapped DELs and DEMs. **(I)** Triple regulatory network of the overlapped DELs, overlapped DEMs, and ferroptosis DEGs. MiRNAs are represented by ellipses, mRNAs by rectangles, and lncRNAs by diamonds. Navy-blue signifies downregulation and red upregulation.

Using miRWalk 2.0 software, we screened 30 ferroptosis DEGs and performed DEG-miRNA analysis. To assure the correctness of the results, crosslinked miRNAs were chosen from the miRWalk and TargetScan databases. Seventeen DEGs and 66 potential miRNA were mined and the network was constructed (Figure 4A and Supplementary Table 3). Based on a *p*-value < 0.05 and | log_2_FC| > 1, 439 DEMs were screened from the GSE66240 dataset, including 55 upregulated and 384 downregulated DEMs (Figure 4B and Supplementary Table 4). Sixty-six mining miRNAs were intersected with DEMs from GSE66240 to identify key miRNAs (Figure 4C). Five overlapped miRNAs were selected (Table 3), and the network was constructed with ferroptosis DEGs (Figure 4D).

**TABLE 3:** Overlapped DEMs.

miRNA ID	Fold change	*P*-value
hsa-miR-143-3p	−1.53	0.040
hsa-miR-143-5p	−3.10	0.003
hsa-miR-378e	−1.44	0.035
hsa-miR-378f	−1.09	0.021
hsa-miR-4644	−1.95	0.008

DELs, differentially expressed miRNAs.

### Further long non-coding RNA interactions and mining

Potential lncRNAs interacting with five key DEMs were screened using Starbase. A network of 570 lncRNAs interacted with five key DEMs (Figure 4E and Supplementary Table 5). Based on a *p*-value < 0.05 and | log_2_FC| > 1, 346 DELs were screened from the GSE122897 dataset, including 109 that were upregulated and 237 that were downregulated (Figure 4F and Supplementary Table 6). A total of 254 mining lncRNAs were intersected with DELs from GSE122897 (Figure 4G). Seventeen overlapped lncRNAs were selected (Table 4), and the network was constructed using key DEMs (Figure 4H).

**TABLE 4:** Overlapped DELs.

lncRNA ID	Fold change	*P*-value
LINC00313	1.14	0.044
LINC00943	−1.73	0.037
DUXAP8	3.02	<0.001
DNAJC27-AS1	−1.38	0.002
AL391244.1	−1.86	0.026
FAM30A	2.01	0.028
AC007405.3	−1.98	0.001
CDKN2B-AS1	1.17	0.028
AC106038.1	−1.12	0.050
PVT1	1.31	0.002
AP000866.2	−1.02	0.033
SLC25A21-AS1	−1.59	0.003
AL356017.1	−1.15	0.008
AC015712.2	1.22	0.018
AC106820.4	−2.33	0.007
AC092384.3	−1.39	0.050
AC011476.2	1.69	0.039

DELs, differentially expressed lncRNAs.

### Construction of the competitive endogenous RNA network

**FIGURE 5:**
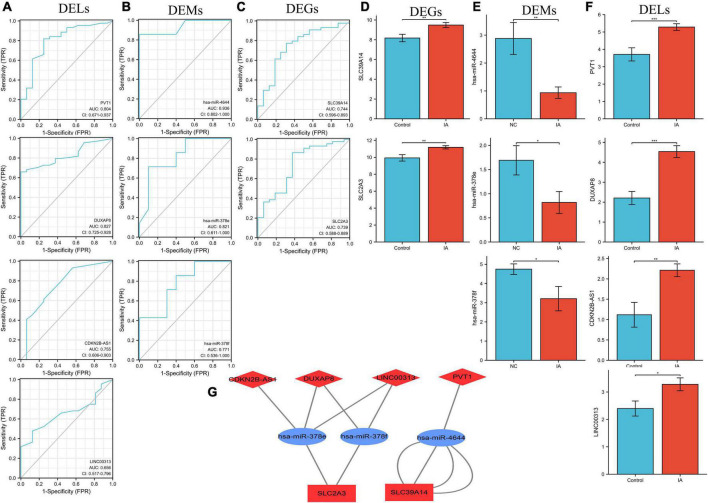
Construction of the key triple regulatory network. ROC analysis of DELs **(A)**, DEMs **(B)**, and DEGs **(C)**. The expression levels of DEGs **(D)**, DEMs **(E)**, and DELs **(F)**. **(G)** The key triple regulatory network components identified by regulation of their expression. MiRNAs are represented by ellipses, mRNAs by rectangles, and lncRNAs by diamonds. Navy-blue signifies downregulation and red upregulation.

In all, 17 overlapped DELs, three overlapped DEMs, and six DEGs were screened to construct a triple regulatory network (Figure 4I). According to expression regulation of DELs, DEMs, and DEGs, a hub triple regulatory network was built using four DELs, three DEMs, and two DEGs (Figure 5G). The areas under the curves (AUCs) for three DELs (PVT1, DUXAP8, and CDKN2B-AS1), three DEMs (hsa-miR-4644, hsa-miR-378e, and hsa-miR-378f), and two DEGs (SLC39A14 and SLC2A3) surpassed 0.7 for all included genes (Figures 5A–C). The expression levels of DELs, DEMs, and DEGs from the triple regulatory network are shown in Figures 5D–F.

**FIGURE 6:**
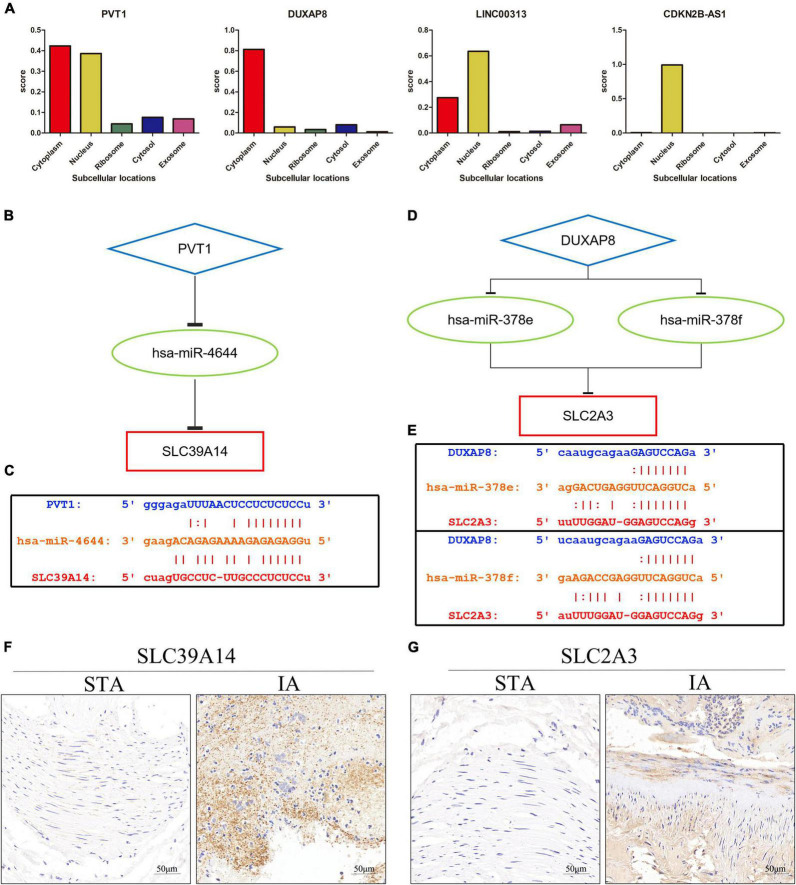
Construction of the ceRNA network. **(A)** The cellular localizations of four DELs (PVT1, DUXAP8, LINC00313, and CDKN2B-AS1). **(B)** A schematic of ceRNA (PVT1-hsa-miR-4644-SLC39A14). **(C)** Base pairing between hsa-miR-4644, PVT1, and SLC39A14 as predicted by StarBase. **(D)** A schematic of ceRNA (DUXAP8-hsa-miR-378e/378f-SLC2A13). **(E)** Base pairing between hsa-miR-378e/378f, PVT1, and SLC39A14 as predicted by StarBase. **(F)** Expression of SLC39A14 in STA and IA tissue. **(G)** Expression of SLC2A3 in STA and IA tissue.

The subcellular localization of the four DELs was investigated using lncLocator because the cellular localizations of lncRNAs determine their functions. The remaining two lncRNAs (LINC00313 and CDKN2B-AS1) were present principally in the nucleus, whereas PVT1 and DUXAP8 were largely confined to the cytoplasm (Figure 6A). These findings suggest that PVT1 and DUXAP8 may serve as ceRNAs that improve SLC39A14 and SLC2A3 expression, respectively. Thus, PVT1-hsa-miR-4644-SLC39A14 ceRNA (Figure 6B) and DUXAP8-hsa-miR-378e/378f-SLC2A3 ceRNA (Figure 6D) networks were constructed. The target sites in the PVT1 and SLC39A14 were predicted to pair with hsa-miR-4644 using StarBase (Figure 6C). Base pairing between hsa-miR-378e/378f, PVT1, and SLC39A14 were also predicted using StarBase (Figure 6E). Immunohistochemistry showed that SLC39A14 (Figure 6F) and SLC2A3 (Figure 6G) were significantly increased in IA tissue.

## Discussion

On the one hand, bioinformatic analyses revealed abnormal expression of ferroptosis-related genes in IA; on the other hand, we found obvious iron deposition in IA tissue samples. This is the first time that IA has been associated with ferroptosis by combined analysis of bioinformatics and pathological samples. We then constructed potential ceRNA regulatory networks using various approaches, providing a possible molecular basis for regulation by ferroptosis of IA formation.

Worldwide, cardiovascular illnesses continue to be the major cause of death. Among them, aneurysms are considered silent killers, and the gravity of their consequences should not be underestimated (Chung et al., 2021). IA is the most common cause of SAH; the multiple pathophysiological alterations that develop after SAH frequently cause irreparable brain damage, complications, and death (Brouwers et al., 1993; Macdonald and Schweizer, 2017; Petridis et al., 2017). Although different treatment options for IA patients have been implemented, most focus on IA development or rupture; they do seek to prevent IA (Arena et al., 2017). A focus on IA formation, progression, and prevention (rather than the molecular mechanisms underlying pathogenesis) may be more beneficial for patients. Many cardiovascular disorders, including atherosclerosis, abdominal aortic aneurysms, and hypertension, are linked to the ceRNA regulatory network (Cai et al., 2020; Yu X. H. et al., 2020; Zhang et al., 2020). Few studies, however, have sought a whole-ceRNA regulation network for IA. As a result, the goal of this study was to create a ferroptosis-related ceRNA triple network for IA. Although there is growing evidence that ferroptosis functions as a regulator in several cardiovascular disorders, any involvement in IA remains unknown.

In this study, IA-related DEGs, DEMs, and DELs were first analyzed from the GEO database using bioinformatic techniques, and 30 ferroptosis-related DEGs were extracted by intersecting DEGs with ferroptosis genes. The PPI network evidenced correlations among ferroptosis-related DEGs. DAB-enhanced Prussian blue staining revealed iron deposition in IA tissues, confirming that ferroptosis may play an important role in IA. Then, the screened ferroptosis-related DEGs, key DEMs, and key DELs were constructed into triple networks, and four DELs, three DEMs, and two DEGs built the hub regulatory networks based on the expression regulation. Finally, this hub regulatory network was subjected to ROC and expression analyses. As the linkages of the ceRNA network are active only in the cytoplasm, we performed subcellular localization analyses of the three lncRNAs in the network. In conclusion, the IA ferroptosis ceRNA networks PVT1-hsa-miR-4644-SLC39A14 and DUXAP8-hsa-miR-378e/378f-SLC2A3 were discovered.

During ferroptosis, oxygen molecules are added to lipids, usually to the polyunsaturated fatty acyl tails of phospholipids, leading to cell death (Hirschhorn and Stockwell, 2019). A variety of diseases have been linked to lipid peroxidation, which, commonly damages the unsaturated lipid moieties of cell membranes and lipoproteins (Romero et al., 1998; Barrera et al., 2008). The reactive oxygen species metabolic pathway played a key role in our function enrichment study. The first stage in IA development is endothelial dysfunction, followed by VSMC phenotypic alteration, extracellular matrix remodeling, and cell death, in turn triggering vascular degeneration, dilatation, and rupture (Starke et al., 2013). Numerous studies have linked inflammation to IA emergence, growth, and rupture (Chalouhi et al., 2012; Hasan et al., 2012). Oxidative stress, attributable to increased free radical formation and/or decreased free radical scavenging, triggers endothelial dysfunction, immune cell infiltration, and VSMC proliferation and migration (Griendling and FitzGerald, 2003; Canes et al., 2021). After pyuvate kinase muscle isozyme 2 activation and lipid peroxidation, T lymphocyte-derived extracellular vesicles exacerbate abdominal aortic aneurysms (Dang et al., 2022). Numerous studies have found that smoking is an independent risk factor for IA, and cigarette extract specifically-triggered ferroptosis of VSMC raises the possibility that ferroptosis may contribute to the pathogenesis of aneurysms (Meschia et al., 2014; Bakker et al., 2020; Sampilvanjil et al., 2020). Few previous studies have investigated the role of ferroptosis in IA. We used bioinformatic analyses and tissue staining to discover abnormal expression of ferroptosis-related genes and iron deposition in IA tissues; ferroptosis seems to play an important role in IA.

We identified two ceRNA networks, PVT1-hsa-miR-4644-SLC39A14 and DUXAP8-hsa-miR-378e/378f-SLC2A3, which may be implicated in the IA disease process. And we verified the expression of SLC39A14 and SLC2A3 in IA by IHC, which further ensured the reliability and accuracy of the results.

SLC39A14, also known as ZIP4, is an important metal transporter that plays an important role in the regulation of metal ions such as manganese (Winslow et al., 2020). Increased levels of SLC39A14 protein on the cell membrane contribute to unstable iron accumulation in skeletal muscles, which in turn lead to the activation of ferroptosis (Ding et al., 2021). Decreased SLC39A14 expression in liver significantly reduces iron accumulation, thereby reducing liver fibrosis mediated by ferroptosis (Yu Y. et al., 2020). These results imply that SLC39A14 may also be crucial in terms of iron control. Although plasmacytoma variant translocation 1 (PVT1) is recognized as an oncogene involved in carcinogenesis, the gene also plays a role in ferroptosis control (Bohosova et al., 2021; He Y. et al., 2021). PVT1 controls ferroptosis associated with cerebral ischemia/reperfusion (I/R) via miR-214-mediated regulation of TFR1 and TP53 expression, while ketamine regulates ferroptosis in hepatoma cells via lncRNA PVT1 (Lu et al., 2020; He G. N. et al., 2021). Hsa-miR-4644 acts as a potential interacting miRNA for SLC39A14 and PVT1; the PVT1-hsa-miR-4644-SLC39A14 ceRNA network may serve as a potential regulatory axis for ferroptosis.

Glucose transmembrane transport are performed by SLC2A3, a membrane transporter that belongs to the solute carrier family (Gao et al., 2021). Lymphoid-specific helicase can inhibit ferroptosis in lung cancer cells, and LSH knockout decreases SLC2A3 and induces ferroptosis, which implies that SLC2A3 may be involved in inhibiting ferroptosis (Jiang et al., 2017). Double homeobox A pseudogene 8 (DUXAP8), as a novel, long noncoding RNA, is associated with a number of cancers, including liver, colorectal, bladder, oral, ovarian, lung, and pancreatic tumors (Xue et al., 2021). However, it is rarely studied in ferroptosis and, whether the DUXAP8-hsa-miR-378e/378f-SLC2A3 ceRNA network we constructed is involved in the regulation of ferroptosis, remains to be verified.

Although the ceRNA-based PVT1/SLC39A14 and DUXAP8/SLC2A3 axes appear to be potential ferroptosis-regulating axes in IA, our work had certain limitations. First, experimental data on the binding affinities of lncRNAs, miRNAs, and mRNAs identified in the databases are required. Second, further research is required to confirm the functions and modes of action of the PVT1/SLC39A14 and DUXAP8/SLC2A3 axes in IA.

## Conclusion

In summary, we constructed two ceRNA pathways (PVT1-hsa-miR-4644-SLC39A14 and DUXAP8-hsa-miR-378e/378f-SLC2A3) using IA information; both are potentially linked to ferroptosis. We hope that our findings will aid future in-depth research.

## Data availability statement

The original contributions presented in this study are included in the article/Supplementary material, further inquiries can be directed to the corresponding authors.

## Ethics statement

Human IA and STA tissue samples were obtained from the First Affiliated Hospital of Nanchang University. The First Affiliated Hospital of Nanchang University’s Ethics Committee gave its approval for this study.

## Author contributions

HZ and YZ: conceptualization. HZ and WZ: methodology. JT: software. ZWa, ZWu, and ZZ: validation. YZ: formal analysis and supervision. HZ: investigation, data curation, and writing—original draft preparation. ZWa: resources. ML: writing—review and editing and project administration. JT and WZ: visualization. YZ and ML: funding acquisition. All authors read and agreed to the published version of the manuscript.

## Footnotes

1
http://www.zhounan.org/ferrdb/

2
https://metascape.org/

3
https://string-db.org

4
http://mirwalk.umm.uni-heidelberg.de/

5
https://starbase.sysu.edu.cn/

6
https://lncipedia.org

7
http://www.csbio.sjtu.edu.cn/bioinf/lncLocator/
