# Supplementary figures and images for: Bioinformatics analysis constructs potential ferroptosis-related ceRNA network involved in the formation of intracranial aneurysm
Source: Front Cell Neurosci. 2022 Oct 13;16:1016682. doi: 10.3389/fncel.2022.1016682 (PMC9612944; doi:10.3389/fncel.2022.1016682)

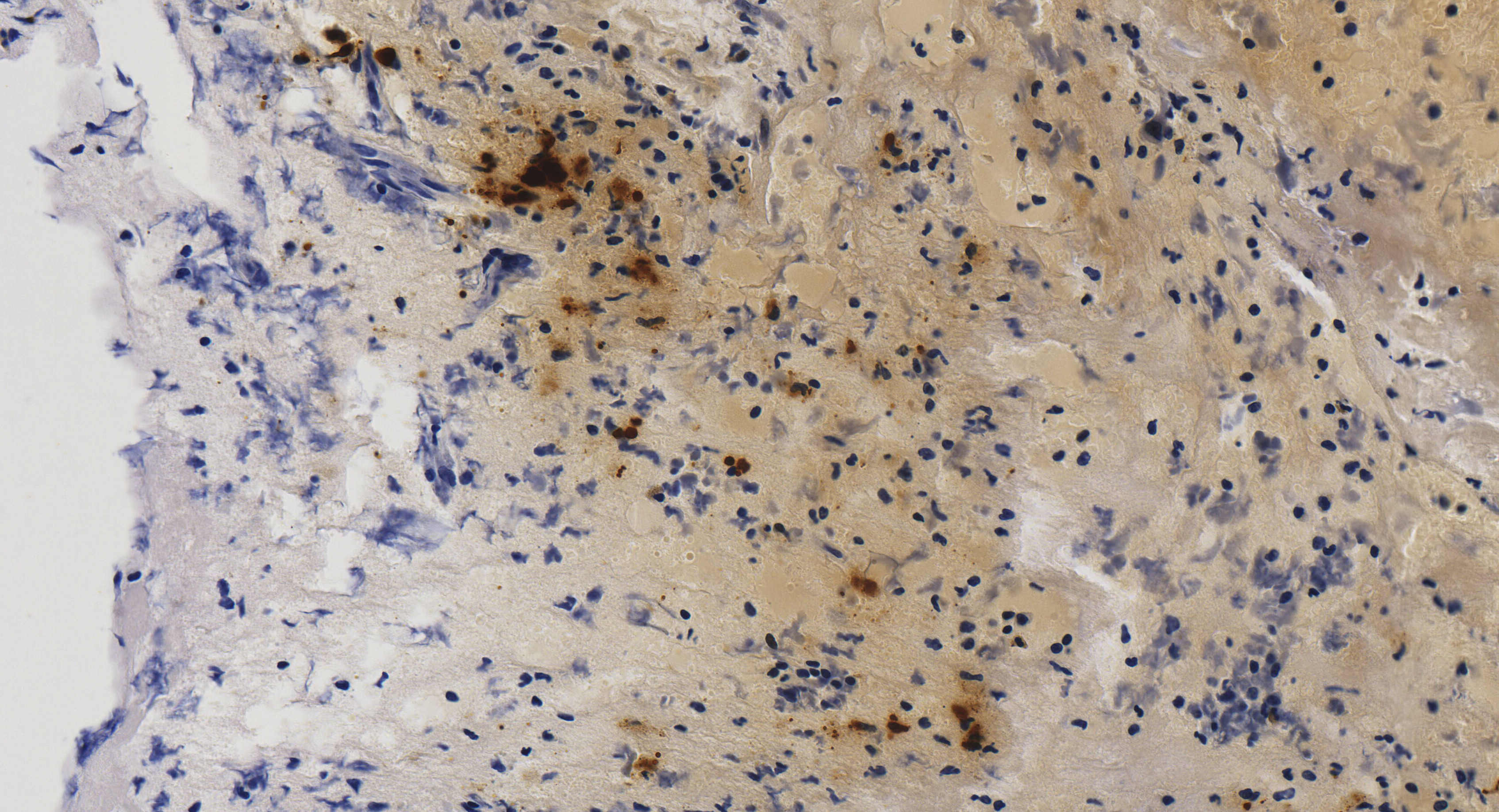

Supplement: Supplementary file 7 [file Data_Sheet_1.ZIP › Raw data/DAB image/IA.tif]

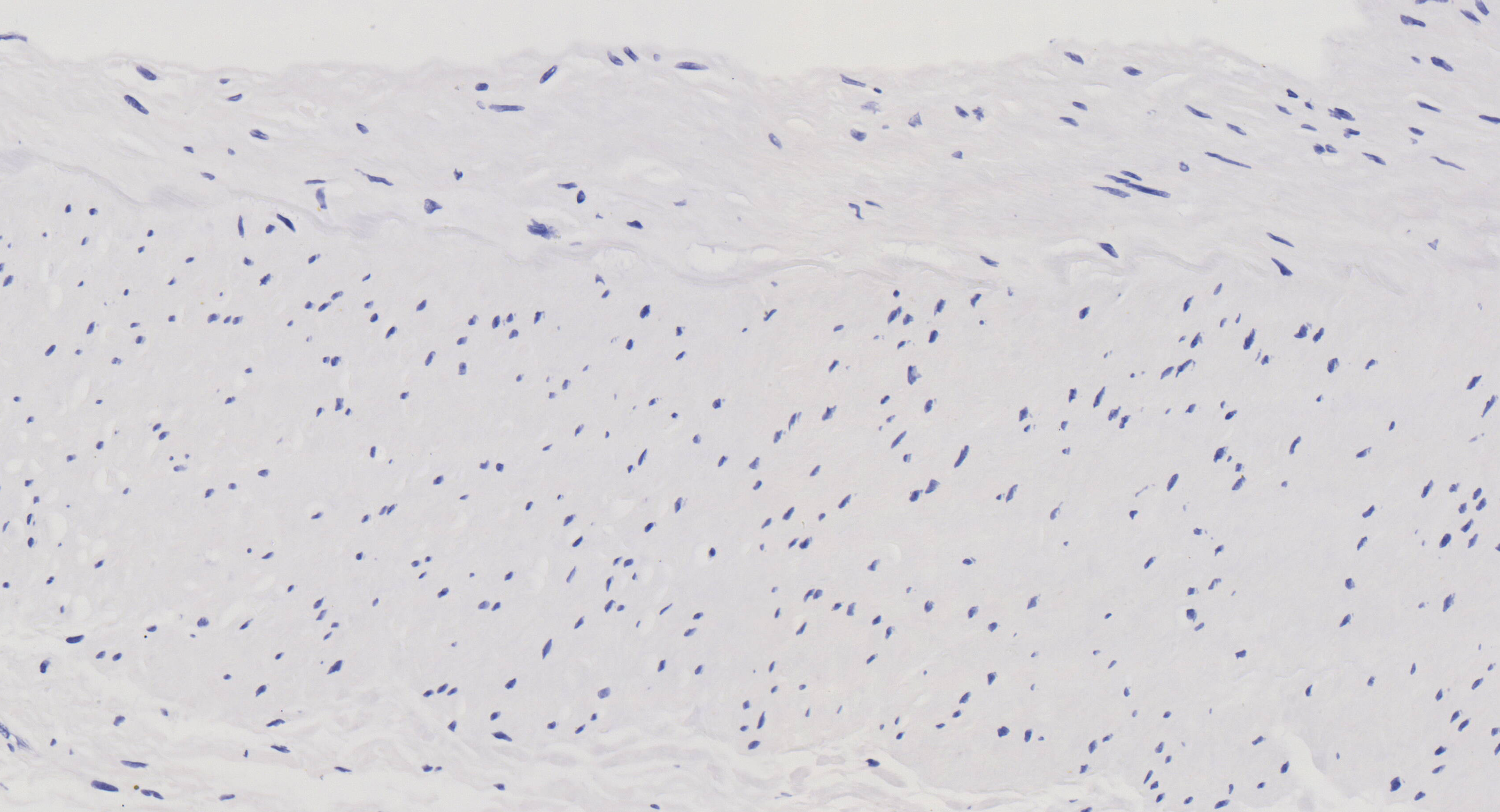

Supplement: Supplementary file 7 [file Data_Sheet_1.ZIP › Raw data/DAB image/STA.tif]

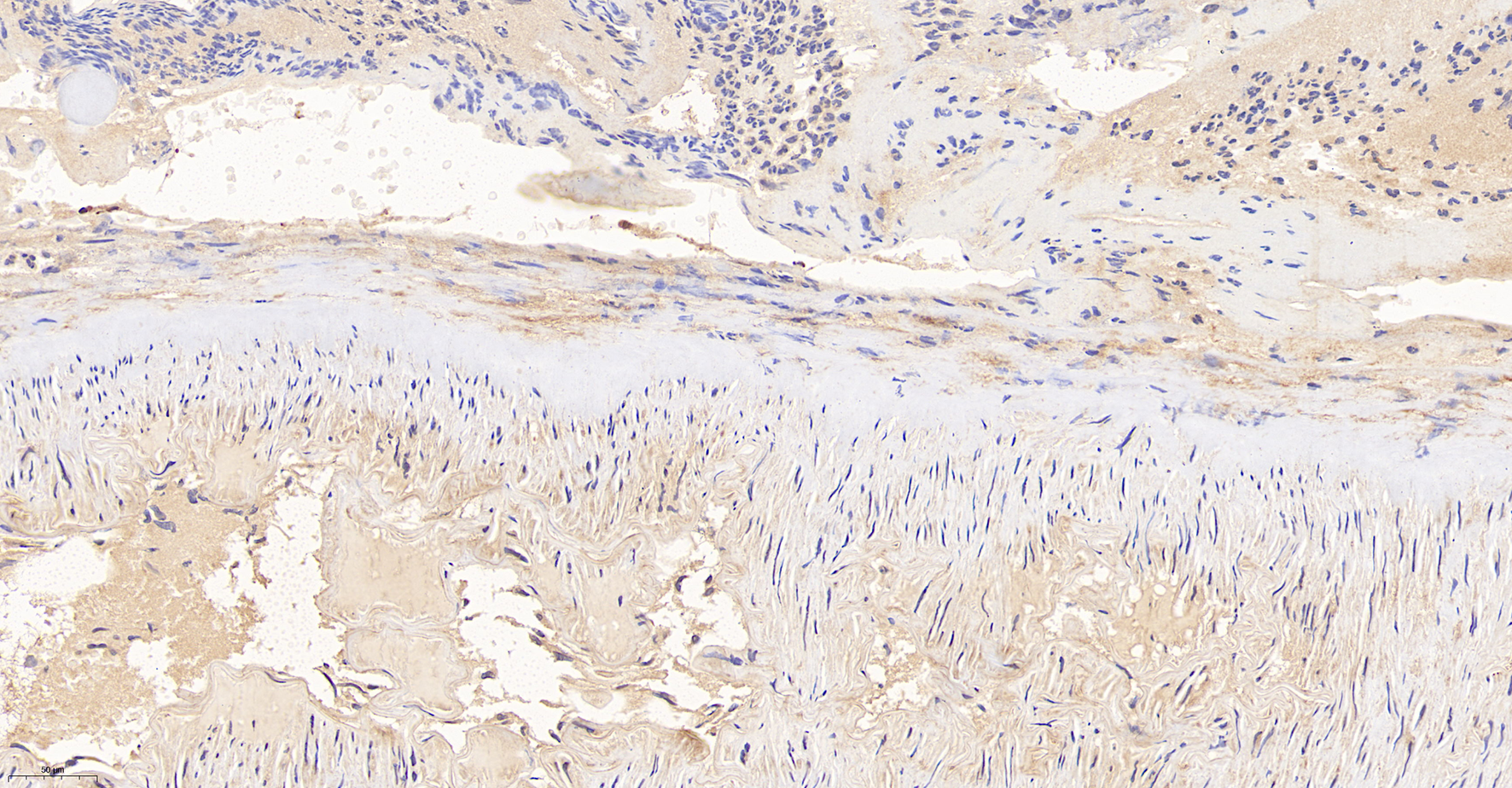

Supplement: Supplementary file 8 [file Image_1.TIF]

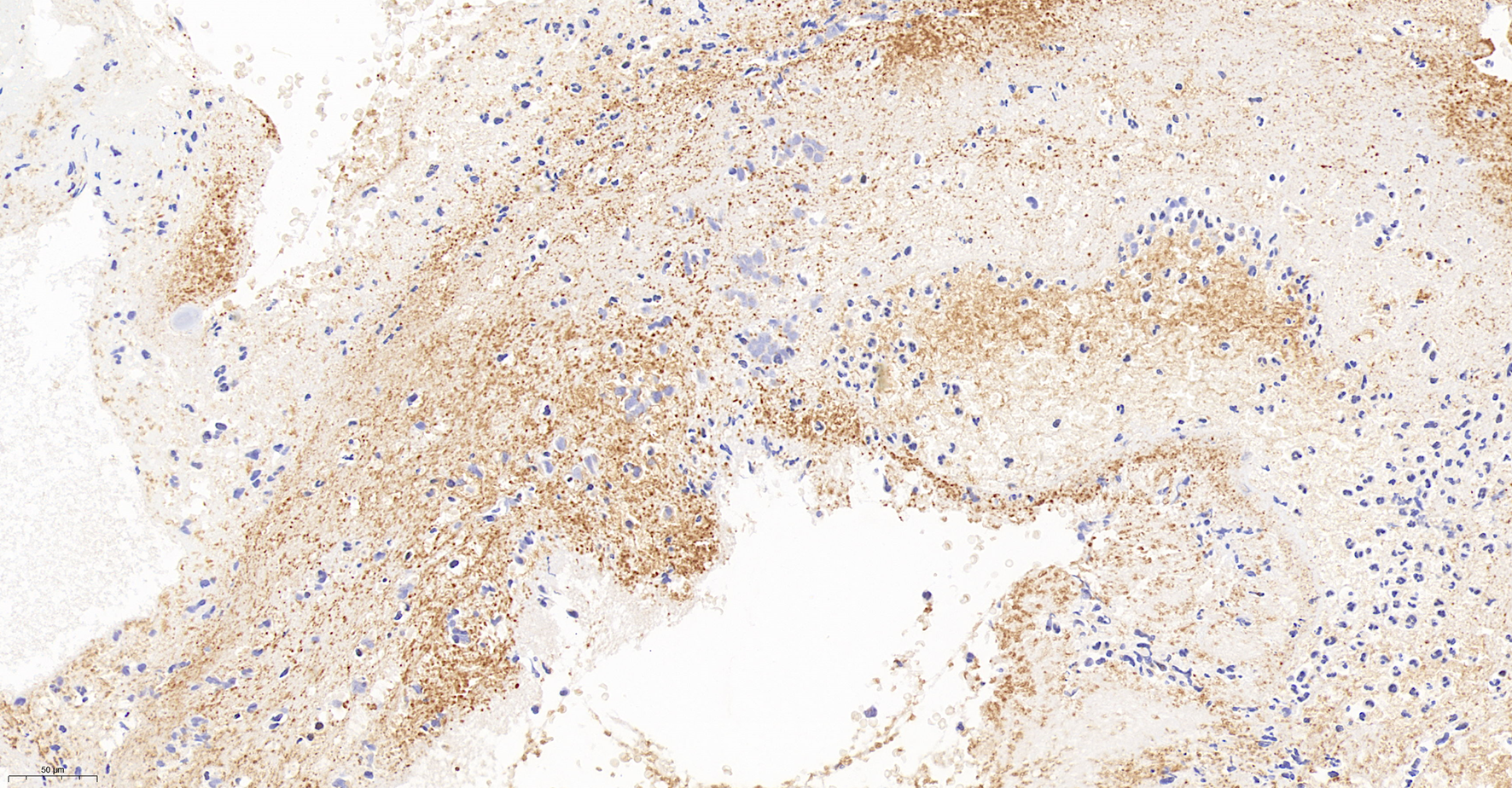

Supplement: Supplementary file 9 [file Image_2.TIF]

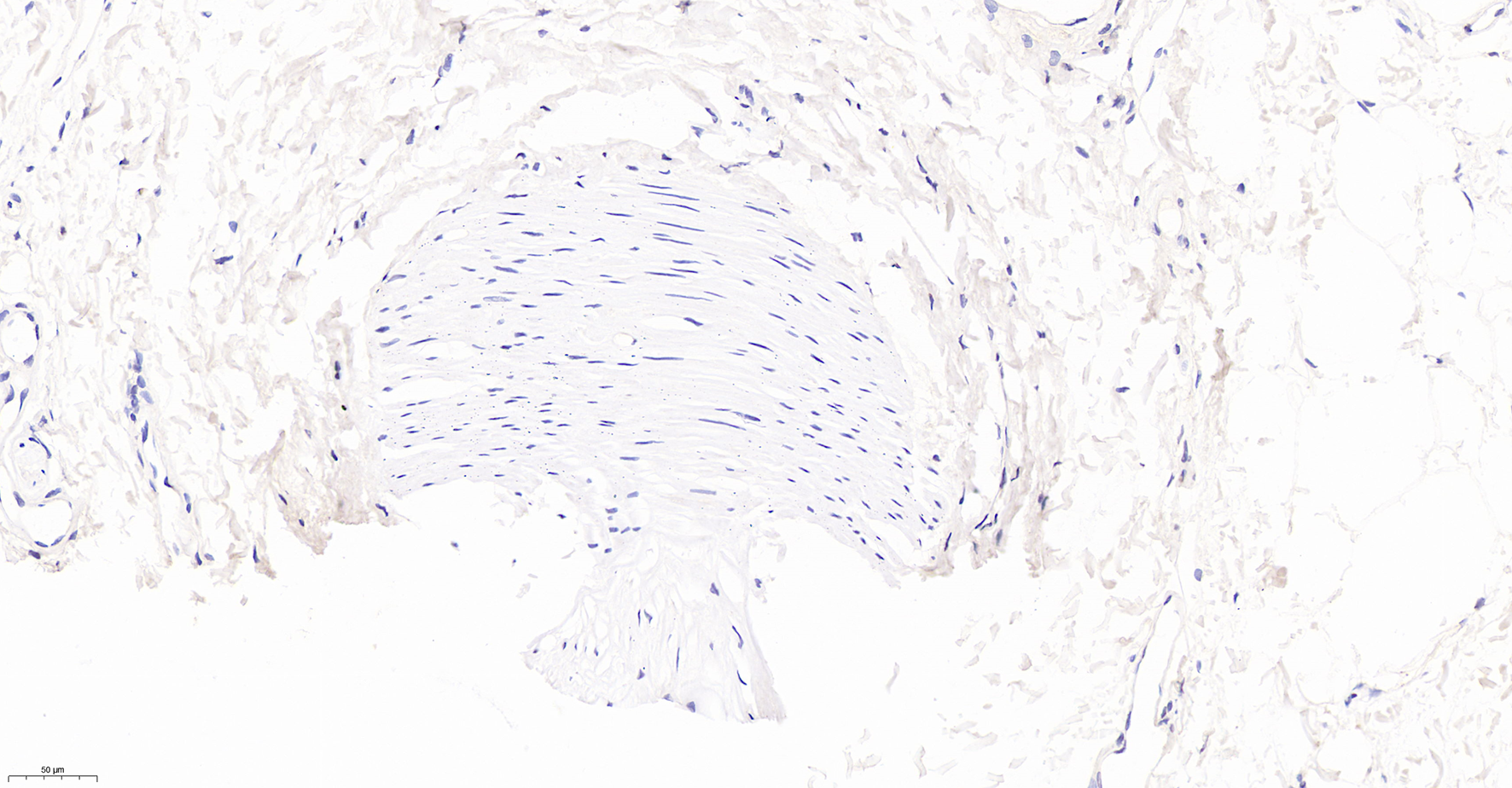

Supplement: Supplementary file 10 [file Image_3.TIF]

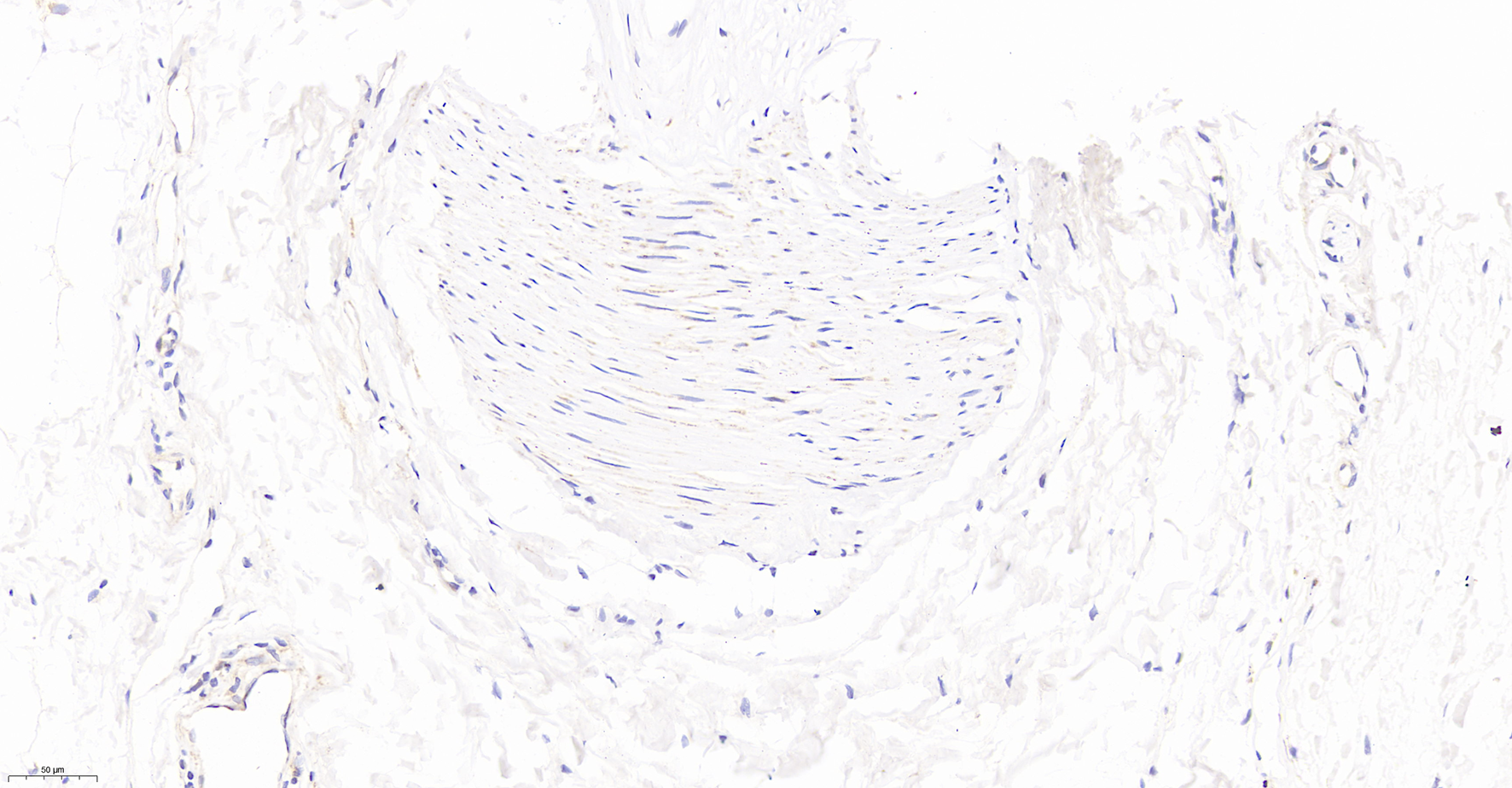

Supplement: Supplementary file 11 [file Image_4.TIF]
